# The COVID-19 global challenge and its implications for the environment – what we are learning

**DOI:** 10.14324/111.444/ucloe.000008

**Published:** 2020-05-12

**Authors:** Francesco Aletta, Dan Osborn

**Affiliations:** 1 Research Associate, Institute for Environmental Design and Engineering, University College London, 2 Taviton Street, London WC1H 0BT, UK; 2 Chair of Human Ecology, Department of Earth Sciences, University College London, 2 Taviton Street, London WC1H 0BT, UK

During the last months, the coronavirus (COVID-19) pandemic has changed the lives of many and had a more or less direct effect on virtually everyone on the planet. This international public health emergency has endless socio-economic ramifications and will have long-lasting consequences [1]. It triggered unprecedented responses from governments around the world, which included implementing strict social distancing and stopping every non-essential productive activity and the related movements of people and goods.

This rapid change is already showing tangible effects in the environmental domain, where the vast majority of emissions associated with world-wide human agency is now suddenly on hold, possibly for the very first time in post-industrial history. Changes are happening in different environmental dimensions and at different scales. Their implications probably have not been fully understood yet, and it is just as difficult to predict what will happen when the current constraints are removed and how societies around the world will collectively recover from this.

To some extent, this pandemic crisis led us to a condition environmental activists were not able to achieve in decades, hard as they tried to lobby ‘green’ policies; that is, a dramatic drop in – inter alia – consumption of goods and services, general volumes of human activity, local commuting, as well as domestic and international travel; and, consequently, a sensitive reduction of environmental pollution at a global level.

Looking at environmental stressors, air pollution and traffic noise are typically ranked as the first two in terms of impact on public health and disability-adjusted life-years (DALYs) [2]. Preliminary reports from research centres and governmental agencies around the world show that both have significantly dropped since lockdown measures were progressively implemented in different countries.

Data from Paris [3] show that since the enforcement of the lockdown on French territory on March 17^th^, 2020 an average reduction of 7.6 dB(A) (L_den_) was observed on the Paris road network, with noise emission reductions ranging between 60% and 90%. Aircraft noise emissions for the Paris Charles De Gaulle airport dropped by an impressive 21.5 dB(A) (L_den_) with air traffic completely blocked, also leading to related noise complaints fading out. Similarly, data from Barcelona [4] show general decreases in noise pollution levels as high as 9 dB one week after the lockdown implementation on March 14^th^, 2020, with a further reduction of 2 dB during the following week. In terms of air pollution, in Central China alone nitrogen dioxide (NO_2_) emissions dropped by 30%, while carbon dioxide (CO_2_) emissions decreased by 25% in China and on average by 6% globally [5]. Such scenarios seem to be common to both industrialised and industrialising countries and predictably be more noticeable in urban contexts.

## A wider context of this global challenge

We share our planet with a range of other organisms, some of which have shaped the planet’s history (e.g. blue-green algae or cyanobacteria), are shaping its present (that is us through greenhouse gas emissions) and will shape its future (us again). Some of these organisms cause disease in humans and wildlife. The evolution of at least some disease organisms or their vectors is intertwined with the nature of human societies and their activities (e.g. influenza strains can develop via a complex mechanism that involves birds and pigs as well as humans). New human and animal diseases can arise when species barriers are crossed, or environmental conditions become suitable for diseases organisms and their vectors to spread.

The role of the environment in disease has been known for a long time. In Medieval and later times, links between health and wellbeing and fossil fuel use were recognised and such practices banned in London on punishment of death (people still kept burning the coal despite this) – and it was not until the UK Clean Air Acts (of the 1950s) that these practices really began to stop. In Roman times sliver smelting near Rome was prohibited when the wind was in the wrong direction because of the fall in urban air quality that smelting metal ores caused.

Often diseases have been dealt with mainly as a medical or public health issue and strategies and technologies for disease management have engaged with environmental matters only in so far as the behaviour of the disease organism or its vector may be affected by variables such as temperature or rainfall (e.g. in diseases like bluetongue [6]; and dengue [7]). Also we know the value of analysing disease outbreaks from geospatial perspectives. This is how John Snow cracked the links between different water sources and a cholera outbreak in mid-19^th^ century London. More recently, similar approaches using more modern technologies have illustrated the interlinkages between social, environmental and disease factors in the case of typhoid in Kathmandu, Nepal [8].

What needs to advance now is our knowledge of how the environment itself can lead to disease (as it does when areas flood and people suffer mental health problems including post-traumatic stress disorder (PTSD)) [9] – findings that are progressively being incorporated into guidance on estimating flood damages [10] and how environmental factors – often when extreme events occur – can cause not only outbreaks of diseases such as cholera but also sufficient loss of community cohesion and resilience that people become susceptible or vulnerable to other health problems.

Despite what we know already we still do not know enough and these issues affect almost every aspect of humanity’s interactions with the environment – thus, for example, the call in Kovats and Osborn [11] for more research and knowledge synthesis on the links between climate change and disease which was partly addressed in [12].

Now, in COVID-19 we have another example of a disease–environment interaction. The issues begin with the original source of the disease (possibly wildlife eaten by people) then range through how the pandemic’s consequences are affecting the environment and our impact on it. This may provide some of the clearest evidence yet of the impact our globalised economy and people’s daily activities have on the environment. This is an unprecedented opportunity but one from which lessons need to be both learned and acted on so that we might see ways of running our society and economy that has less of an impact on the environment and increases overall human and planetary resilience.

There is already pressure to reopen the economy to recover from the damage the disease has done to it and the public and private finances. This pressure could lead to more damage to human health and a worse environment if not managed carefully. And all this at a time when environmental improvements are needed: for example, on climate change and on biodiversity and to meet many of the Sustainable Development Goals. To achieve the environmental improvements a lot of innovation and a high level of investment is needed such that governments must commit wholeheartedly to them and bring the private sector and its financiers along with them. This will be particularly true if negative emissions technologies have to be deployed.

Thus, we face a three-way challenge.

All of this suggests a great deal of debate is needed on how people relate to their environment and how the use of the environment affects all the species and habitats on this planet including those that cause disease. Economists are themselves already starting to consider some of these issues [13]. This debate could inform how we best move forward after the current pandemic is controlled and might help indicate how best a globalised world can run its economic system in the best interests of people’s well-being.
